# Biochemical Characterization of a Polysialyltransferase from *Mannheimia haemolytica* A2 and Comparison to Other Bacterial Polysialyltransferases

**DOI:** 10.1371/journal.pone.0069888

**Published:** 2013-07-26

**Authors:** Theresa Lindhout, Cynthia R. Bainbridge, Will J. Costain, Michel Gilbert, Warren W. Wakarchuk

**Affiliations:** 1 Department of Molecular and Cellular Biology, University of Guelph, Guelph, Ontario, Canada; 2 Human Health Therapeutics, National Research Council Canada, Ottawa, Ontario, Canada; 3 Department of Chemistry and Biology, Ryerson University, Toronto, Ontario, Canada; La Trobe University, Australia

## Abstract

Polysialic acids are bioactive carbohydrates found in eukaryotes and some bacterial pathogens. The bacterial polysialyltransferases (PSTs), which catalyze the synthesis of polysialic acid capsules, have previously been identified in select strains of *Escherichia coli* and *Neisseria meningitidis* and are classified in the Carbohydrate-Active enZYmes Database as glycosyltransferase family GT-38. In this study using DNA sequence analysis and functional characterization we have identified a novel polysialyltransferase from the bovine/ovine pathogen *Mannheimia haemolytica* A2 (PST_Mh_). The enzyme was expressed in recombinant form as a soluble maltose-binding-protein fusion in parallel with the related PSTs from *E. coli* K1 and *N. meningitidis* group B in order to perform a side-by-side comparison. Biochemical properties including solubility, acceptor preference, reaction pH optima, thermostability, kinetics, and product chain length for the enzymes were compared using a synthetic fluorescent acceptor molecule. PST_Mh_ exhibited biochemical properties that make it an attractive candidate for chemi-enzymatic synthesis applications of polysialic acid. The activity of PST_Mh_ was examined on a model glycoprotein and the surface of a neuroprogenitor cell line where the results supported its development for use in applications to therapeutic protein modification and cell surface glycan remodelling to enable cell migration at implantation sites to promote wound healing. The three PSTs examined here demonstrated different properties that would each be useful to therapeutic applications.

## Introduction

Polysialic acid (PSA) plays a crucial role on the surface of eukaryotic neuronal cells and neuro-invasive pathogens like *Neisseria meningitidis*. In mammals PSA is presented as a homopolymer of α2,8-linked *N*-acetyl-neuraminic acid (Neu5Ac) residues on a small number of proteins of which the major carrier is the neural cell adhesion molecule (NCAM). It has been shown that the PSA on NCAM modulate cell-cell interactions through anti-adhesive properties during development (reviewed in [1]). The NCAM molecule has been implicated in numerous normal and pathological processes, including mammalian development, neuronal plasticity, and tumour metastasis [2]. PSA is also found on CD36 [3], as well as on the recently described SynCAM [4]. Recently the PSA portion of NCAM was shown to have a very important role in the brain since removal of the polysialylation resulted in severe brain defects and precocious death [5], [6]. In addition, changes to the amount and chain length of PSA may influence psychiatric disorders through impaired binding to brain-derived neurotrophic factor and dopamine, which are linked to schizophrenia and other psychiatric disorders, such as depression and bipolar disorder [7].

In bacteria three PSA variants are produced as capsular polysaccharides (for a review of sialic acid biosynthesis see [8]). Neuro-invasive human pathogens *Escherichia coli* K1 and *N. meningitidis* group B as well as the bovine/ovine pathogen *Mannheimia haemolytica* A2 produce a capsule composed of homopolymer of α2,8-linked Neu5Ac residues [9], [10]. Additionally, *N. meningitidis* group C produces a capsule with a homopolymeric α2,9-linked structure [11], whereas *E. coli* K92 produces a capsule with alternating linkages of α2,8/α2,9-linked Neu5Ac [12]. It has been established that the mimicry of PSA by these meningitis-causing strains of *E. coli* and *N. meningitidis* allows them to avoid detection by the host immune system, thereby contributing to their pathogenesis [13].

Polysialyltransferases (PSTs) are membrane-associated enzymes that catalyze the transfer of Neu5Ac from the activated sugar donor CMP-Neu5Ac to the non-reducing end of the growing polymer. In mammals the PSA chain is synthesized by two Golgi-resident enzymes, ST8Sia2 and ST8Sia4, which have a strong specificity for their protein target(s) [14], [15]. These enzymes are found in the Carbohydrate-Active enZYmes database (CAZy) family GT-29 [16], along with all other mammalian sialyltransferases. In bacteria, genes encoding PSTs in *E. coli* K1 and K92 as well as from *N. meningitidis* group B and C are located in the capsule biosynthesis locus of the respective organisms [17], [18]. These enzymes have been characterized in recombinant form [19], [20], [21], [22], [23] and grouped into CAZy family GT-38, which consists exclusively of bacterial PSTs. Bacterial PSTs function at the cytoplasmic side of the inner membrane next to the capsule export machinery, which has posed a challenge for expression and analysis of recombinant forms of these peripheral membrane enzymes.

Recently the PSA polysaccharide has been discovered to have a function in biotechnology where it is used as a chemical means of improving therapeutic protein half-life [24], [25]. This chemical conjugation is not the only way of attaching PSA on therapeutic proteins; we have also shown it can be added enzymatically to existing *N*-linked glycans using the bacterial PST enzyme from *N. meningitidis* group B [26]. Since the site-specific PSA addition could be applied to many recombinant therapeutic glycoproteins it would be advantageous to improve the PST enzyme's profile, in particular the specific activity and purity.

The animal pathogen *M. haemolytica* A2 has a PSA capsule and has been previously tested for polysialyltransferase activity [27], although to our knowledge the enzyme has not been purified or characterized. Our primary objective in this study was to identify the gene encoding this enzyme and characterize the enzyme in recombinant form. We further expanded this analysis to include a side-by-side comparison with two other recombinantly-expressed PSTs from *N. meningitidis* group B and *E. coli* K1. Although the latter two enzymes have been previously characterized by us and others, there is significant variation in their performance depending on the expression construct, purification method, and activity assay used. Furthermore, an improved purification method has resulted in significantly improved enzyme performance [26]. Characterizing the three enzymes in parallel allowed us to evaluate their utility as tools for glycoprotein modification, and for other applications of PSA synthesis.

## Materials and Methods

### Analysis of the *M. haemolytica* A2 genome sequence

All molecular biology methods were performed according to manufacturer's instructions, where appropriate. Genomic DNA from *M. haemolytica* A2 (ATCC 29694) was isolated using the DNeasy Tissue kit (Qiagen Inc., Toronto, Canada). Genomic DNA was amplified using Phusion polymerase (New England Biolabs, Pickering, Canada) with primers based on the sequences of *M. haemolytica* A1 capsule biosynthetic genes *wzf* and *phyB*, which were expected to be conserved (Forward 5′-CAGTTGGAATAATTACAGTTAGCCA and Reverse 5′- CTTTTTGCTCACTTTCTAACCAACG). The ∼8.2 kb PCR amplicon was sequenced using primer walking followed by submission of the sequence to GenBank (FJ217347). The sequence of *pst_Mh_* was confirmed following the subsequent publication of the *M. haemolytica* A2 genome sequence (NZ_ACZY010000016.1, COK_0618) [28].

### Cloning of bacterial polysialyltransferases

PST_Mh_ was amplified as a full-length and a Δ20 N-terminal truncation using forward primers 5′-GTAGGTTAACATATGATTAAGACAATAAAGAAATTATTAGTATC and 5′-CTTTCAGGATCATATGTTTTTAAAATTTCATTTAGCAGAAG, respectively, with the following reverse primer 5′-CGTTGGGTCGACTTACTATATTTCATTTGTATTATTTAATATTC. A Δ15PST_Ec_ amplicon was generated by amplification of the PST_Ec_ DNA sequence with primers 5′-GGCTACGCATATGAATCCAATTGGGTTTTTCCGT and 5′-CCGAGCCGTCGACCTATTACTCCCCCAAGAAAATCCTT. PCR amplicons were then digested *Nde*I and *Sal*I restriction enzymes (underlined in primer sequence) (New England Biolabs, Pickering, Canada) followed by ligation with T4 DNA ligase (Roche Diagnostics, Laval, Canada) into the expression vectors pCW or pCWMalE-thrombin, where appropriate. MalE-PST_Ec_, MalE-PST_Nm_, and MalE-Δ19PST_Nm_ were expressed from constructs previously generated by our laboratory [22], [26]. PST constructs were then transformed into *E. coli* AD202 (*E. coli* Genetic Stock Center, CGSC 7297) for expression.

### Expression and Purification of Recombinant Polysialyltransferases

The various PST constructs were expressed and purified using the conditions previously described for MalE-glycosyltransferase fusion proteins [26], [29]. Briefly, cells were grown in 2YT media at 37°C until the culture reached OD_600_∼0.5 followed by induction with 0.5 M IPTG and overnight growth at 25°C. Cells were harvested by centrifugation and stored at −80°C. To extract the enzyme the cells were thawed and re-suspended in PBS buffer followed by lysis with an Emulsiflex-C5 (Avestin) and centrifugation (27 000× *g*). Soluble protein was purified using a 5 mL HiTrap Heparin HP column (GE Healthcare, Baie d'Urf'é, Canada) with a linear gradient of 0–60% B using the following buffers: A, PBS pH 7.4; B, PBS pH 7.4+2 M NaCl. Fractions containing the purest enzyme were pooled together and stored in the column elution buffer. Attempts to desalt the protein resulted in partial loss of activity, so the preparations were used without desalting and therefore contained approximately 500 mM NaCl.

### Polysialyltransferase Activity Assays on Small Molecule Acceptors

The activity of the PSTs was determined by using synthetic monosialyl-lactosyl (GM3), disialyl-lactosyl (GD3), and trisialyl-lactosyl (GT3) ganglioside analogues (glycan portion only) coupled to the fluorophore 6-(5-fluoresceincarboxamido)-hexanoic acid succinimidyl ester (FCHASE) (Invitrogen, Burlington, Canada), which were synthesized as previously described [22]. The standard assay conditions were: 0.5 mM GD3-FCHASE, 50 mM HEPES pH 7.5, 10 mM MgCl_2_, and 10 mM CMP-Neu5Ac at 30°C. Where indicated in the text some of these parameters were changed including the acceptor molecule (GT3-FCHASE), buffer and pH (MES pH 5.5, HEPES pH 7.0, Tris-HCl pH 8.0, Tris-HCl pH 9.5), temperature (25°C, 37°C), and the concentration of CMP-Neu5Ac for kinetic analysis (0.25 mM to 20 mM). The assays were analysed by capillary electrophoresis as previously described [22].

### Polysialyltransferases Activity Assays on Fetuin

The activity of the PSTs was determined on the bovine serum glycoprotein fetuin which had been enzymatically modified so that the *N*-glycans terminated in two Neu5Ac residues (disialyl-fetuin, diSA-Fet) as previously described [26]. The PSTs were incubated with either 1 mg/mL or 3 mg/mL diSA-Fet in the presence of 50 mM sodium phosphate buffer pH 8.0, 10 mM MgCl_2_, and 10 mM CMP-Neu5Ac at 30°C for a period of time indicated in the text. The amount of enzyme used in the reactions (1 mU) was standardized according to the specific activity determined from reactions with GD3-FCHASE. The reactions were stopped using 1× protein sample buffer and analyzed by SDS-PAGE. The presence of PSA was confirmed by performing Western blots probed with anti-PSA antibodies (mAb735, kind gift of Dr. Rita Gerardy-Schan, Hanover Medical School).

### Production of Polysialic Acid on the Surface of PC12 Cells

PC12 cells (CRL-1721, ATCC) were plated at a density of 1×10^6^ cells/well on 12 mm coverslips (Bellco), coated with 25 µg/ml poly-l-lysine, in 24 well sterile culture plates. PC12 cells were grown in DMEM media (containing 2.5% heat inactivated fetal calf serum, 15% heat inactivated horse serum, 100 U/ml penicillin, 100 µg/ml streptomycin) for 24–48 hours prior to treatment. The cells were washed once with serum-free media immediately prior to treatment with the PSTs. The PST reaction was conducted for 1 hour at 37°C in the presence of 5% CO_2_ in a humidified incubator in a 200 µl volume of DMEM containing 5 mM CMP-Neu5Ac and 4 mU PST. Subsequent to the PST reaction, the cells were washed twice with PBS and fixed with 200 µl formalin for 10 min at room temperature. The cells were then washed twice with PBS and stored in PBS at 4°C until use in immunocytochemistry.

### Immunocytochemical Detection of PSA on PC12 Cells

Polysialylation of PC12 cells was determined using α-PSA-NCAM immunocytochemistry. Briefly, fixed cells were blocked in PBS containing 1% BSA and 0.05% Triton X-100 for 30 min at room temperature. The cells were then incubated for 60 min at room temperature with α-PSA-NCAM (1∶200, mouse IgM clone 2-2B, Millipore, MAB5324) in PBS buffer (with BSA and Triton as above). The cells were then washed three times with PBS prior to incubation with goat anti-mouse IgM Alexa488 conjugate (1∶600, Molecular Probes, A-21042) in PBS (with BSA and Triton as above). Thereafter, the cells were washed 3 times with PBS and once with dH_2_O prior to mounting with Vectashield (Vector Labs, Burlingame, CA) containing 5 µg/ml Hoechst 33258. Confocal fluorescent images were obtained using an Olympus Fluoview FV1000 upright microscope with a 40× objective (UPLSAPO, 0.95 NA).

## Results

### Identification of a putative polysialyltransferase from the *Mannheimia haemolytica* A2 genome sequence

The DNA coding sequence for a putative PST was determined by sequence analysis of a region of the *M. haemolytica* A2 genome. Using primers based on the sequences of *M. haemolytica* A1 capsule biosynthesis genes *wzf* and *phyB*, a region of the *M. haemolytica* A2 genome was amplified and the sequence was analyzed using primer walking. A 1.2 kb DNA sequence was identified that encoded a putative protein (henceforth referred to as PST_Mh_) of 401 amino acids long and a predicted molecular weight of 47.7 kDa. An examination of the corresponding amino acid sequence using a BLAST alignment showed that it shared 35% and 31% identity, respectively, with the previously described PST_Nm_ and PST_Ec_, which is at a similar level to the identity reported for the latter two enzymes (33%) [21], [22]. Based on the observed sequence similarity, this gene was cloned and expressed to detect for PST activity.

### Evaluation of Solubility and Activity of Polysialyltransferase Constructs

PST_Mh_ was cloned and expressed as three variants: full length (PST_Mh_), full length with an N-terminal maltose binding protein fusion (MalE-PST_Mh_), and with a 20 amino acid N-terminal truncation fused with MalE (MalE-Δ20PST_Mh_). The truncation of amino acids at the N-terminus to remove a putative trans-membrane domain as well as the addition of MalE has previously been shown to improve the solubility and specific activity of PST [22], [23], [30].

**Figure 1:**
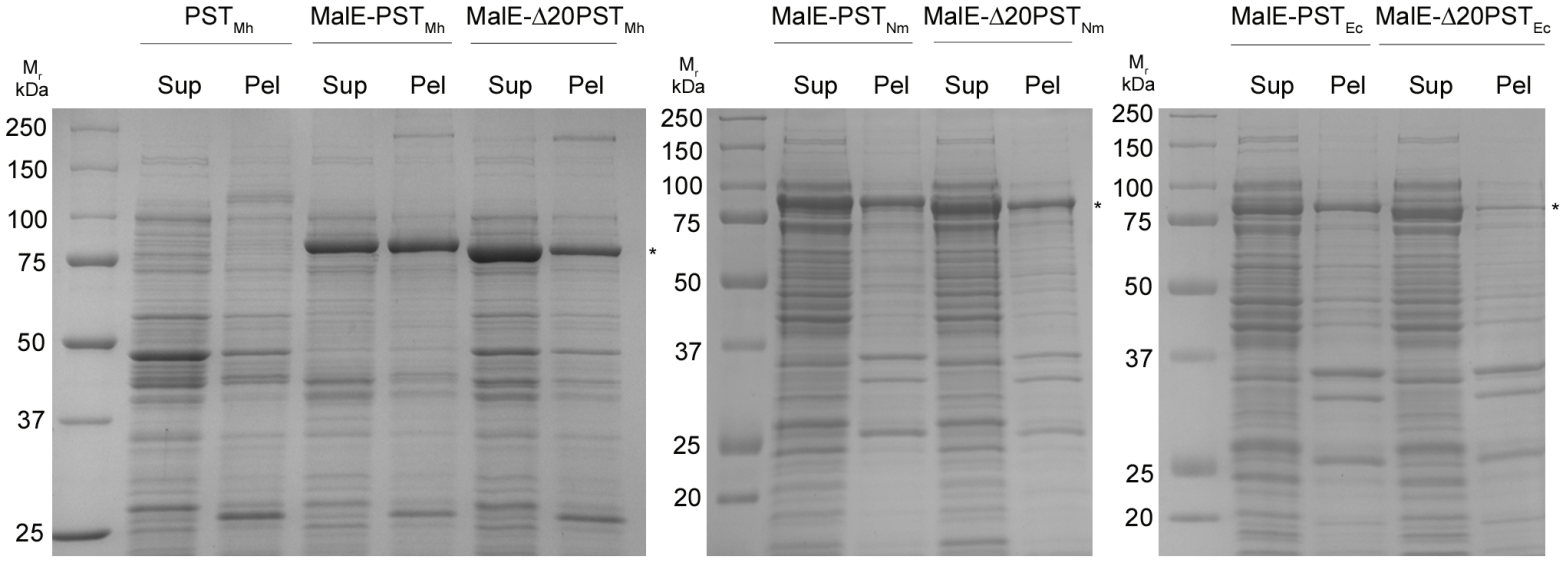
Expression and solubility of PST_Mh_ constructs. The PST constructs were expressed in *E. coli* AD202 followed by cell disruption and centrifugation. The soluble (Sup) and insoluble (Pel) fractions were analyzed by SDS-PAGE and Coomassie Blue staining. Bands corresponding to the predicted sizes of the protein variants were observed (indicated with an asterisk). The increased intensity of the bands of the MalE-fusions indicated increased expression compared to the non-fusion protein. Furthermore, the truncated protein (MalE-Δ20PST_Mh_) variant demonstrated the best solubility.

In order to measure the solubility of the various PST constructs, the specific activities of the soluble and insoluble fractions of the cell lysates were determined using the fluorescent synthetic acceptor molecule GD3-FCHASE followed by detection using capillary electrophoresis (CE). PST activity was detected in both the soluble and insoluble fractions of all construct expressions. The fusion to MalE resulted in approximately a five-fold increase in activity compared to PST_Mh_ (Table 1). Furthermore, the truncation of PST_Mh_ (MalE-Δ20PST_Mh_) resulted in an increase in the proportion of activity in the soluble fraction (86% compared to ∼45–50% for the full-length constructs). In support of these results, qualitative SDS-PAGE analysis indicated that MalE-Δ20PST_Mh_ demonstrated the best combination of expression and solubility of the three constructs (Figure 1). The activity of the soluble and insoluble fractions for the corresponding PST_Nm_ and PST_Ec_ constructs was also examined. Similarly, both MalE-Δ19PST_Nm_ and MalE-Δ15PST_Ec_ demonstrated an increased ratio of soluble activity from 1∶1 to 3∶1 compared to MalE-PST_Nm_ and MalE-PST_Ec_ (Table 1 and Figure 1). Interestingly, the specific activity of the soluble material from the cell lysate was detectably higher for the full-length MalE fusions compared to their truncated counterparts. However, once purified, the activity of the truncated proteins was restored to similar levels as the full-length purified proteins (data not shown). Since the truncated MalE-fusion proteins resulted in the best solubility, expression, and activity they were chosen for further biochemical analysis.

**Table 1:** Comparison of Soluble Activity of PST Constructs^1^.

Enzyme	Supernatant (mU/mg)	Pellet (mU/mg)	% Sup	% Pel
**PST_Mh_**	76.4±15.7	101.1±9.4	43.1%	56.9%
**MalE-PST_Mh_**	439.7±10.4	454.9±80.4	49.2%	50.8%
**MalE-Δ20PST_Mh_**	341.0±4.8	55.3±7.9	86.0%	14.0%
**MalE-PST_Nm_**	351.1±185.9	290.3±135.6	54.7%	45.3%
**MalE-Δ20PST_Nm_**	88.0±42.9	29.9±17.7	74.6%	25.4%
**MalE-PST_Ec_**	169.6±15.2	201.3±1.0	45.7%	54.3%
**MalE-Δ20PST_Ec_**	68.8±9.3	24.2±1.9	74.0%	26.0%

1 Experiments were performed in triplicate (*n* = 3) with mean values and standard error reported.

### Acceptor Molecule and pH Conditions for Optimal Activity

**Figure 2:**
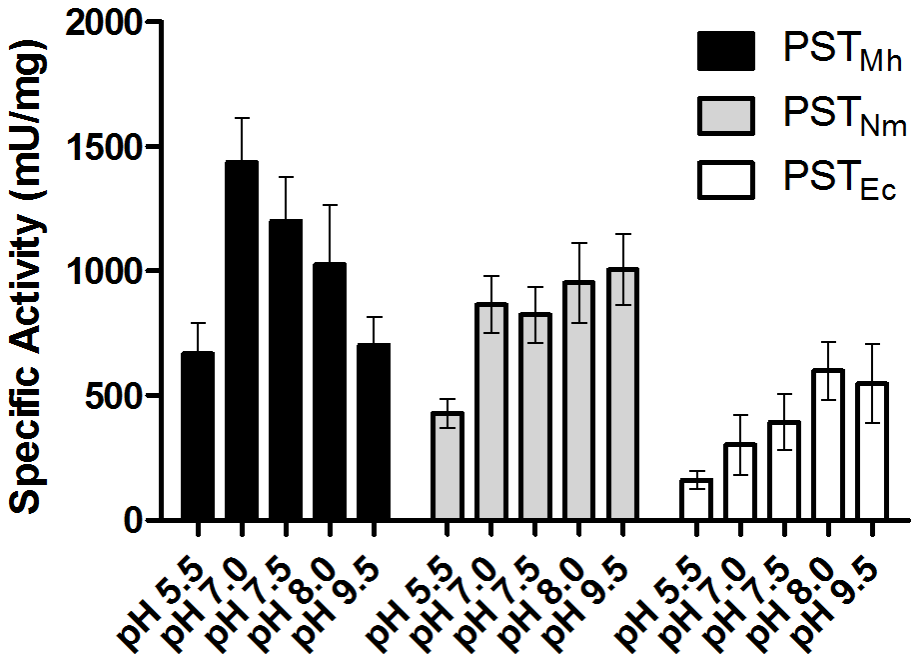
pH optima of PST_Nm_, PST_Ec_, and PST_Mh_. The specific activities of the three bacterial PSTs were compared at different reaction pHs. . Enzymes from three separate purification batches were tested in duplicate (*n* = 6). All enzymes demonstrated activity across the range of pHs tested. PST_Mh_ demonstrated a reaction pH optimum of 7.0. PST_Nm_ and PST_Ec_ demonstrated optimal activity at slightly higher pH (7.5–8.0).

Having established the optimal construct (MalE-Δ20PST_Mh_, hereafter referred to simply as PST_Mh_) we next wanted to evaluate several biochemical properties of the enzyme and compare them to the equivalent constructs of the other two bacterial PSTs (PST_Nm_ and PST_Ec_). We first examined if PST_Mh_ had a preference for a disialyl (GD3-FCHASE) or trisialyl (GT3-FCHASE) acceptor molecule. In the case of both acceptors the specific activities of PST_Mh_ and PST_Nm_ were significantly greater than that of PST_Ec_ (Table 2). PST_Mh_ and PST_Nm_ demonstrated a clear preference (nearly two-fold greater) for GT3-FCHASE, in contrast to PST_Ec_, which has a slight preference for GD3-FCHASE. None of the PSTs demonstrated activity on the monosialyl acceptor molecule GM3-FCHASE (data not shown), which is consistent with published evidence that at least two Neu5Ac residues on the acceptor molecule are required [22], [26]. We next wanted to determine the optimal reaction pH for PST_Mh_ in the range between pH 7.0–pH 8.0, since these pH values would likely be most suitable for downstream applications as they are near physiological pH and also since previous studies have shown that PSTs from *E. coli* and *N. meningitidis* have a broad range of pH tolerance with an optimum at pH 8.0 [22], [23]. We also analyzed reactions at outlying pH values, including pH 5.5, which was expected to be unsuitable for large-scale reactions because of its acidity, as well as a very basic pH at 9.5. PST_Mh_ demonstrated optimal activity at pH 7.0, while still retaining relatively high levels of activity at pH 7.5 and pH 8.0 (Figure 2). Similarly, PST_Nm_ and PST_Ec_ demonstrated activity across a range of pH values, although these enzymes demonstrated higher activity at a slightly more basic pH range from pH 7.5–pH 9.5.

**Table 2:** Biochemical Properties of Bacterial Polysialyltransferases^1^.

	Specific activity^2^		CMP-Neu5Ac Kinetics^2^		
Enzyme	GD3-FCHASE (mU/mg)	GT3-FCHASE (mU/mg)	K_m_ (mM)	V_max_ (µmol/min/mg)	V_max_/K_m_
**PST_Mh_**	1433.4±83.8	2670.8±92.1	1.1±0.05	1519.0±96.2	1380.9
**PST_Nm_**	939.6±222.2	1885.7±173.1	5.7±0.7	1318.5±40.3	251.9
**PST_Ec_**	322.1±56.1	225.5±50.3	4.9±0.7	800.4±112.4	162.4

1 Experiments were performed in triplicate (*n* = 3) with mean values and standard error reported.

2 Experiments were performed with enzymes from three separate purification batches in duplicate (n = 6) with mean values and standard error reported.

### Stability of the Enzymes During a Timecourse

**Figure 3:**
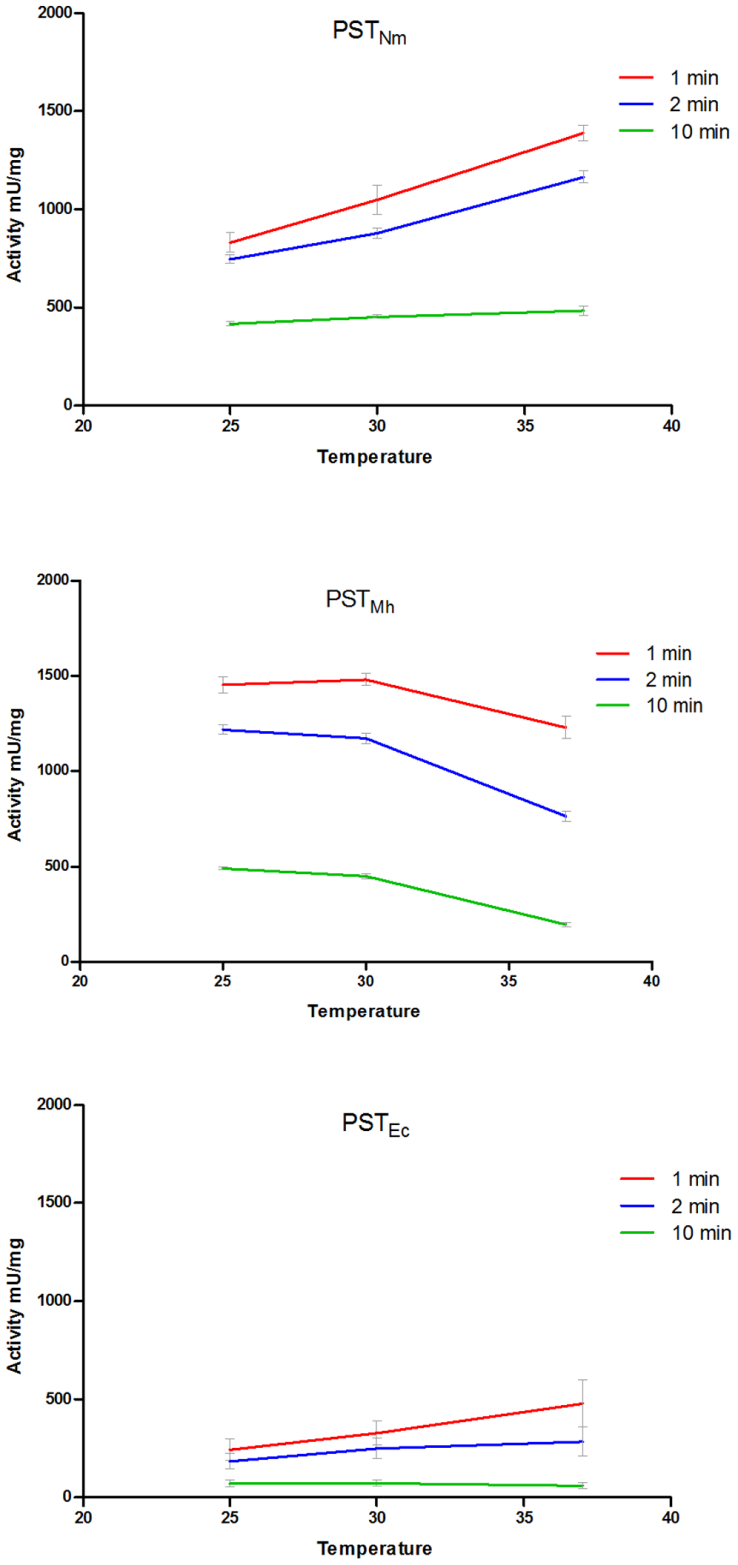
Stability and thermolability of PST_Nm_, PST_Ec_, and PST_Mh_. The specific activities of the three bacterial PSTs were monitored during a two-hour time course at three different reaction temperatures (25°C, 30°C, and 37°C). Both PST_Nm_ and PST_Ec_ demonstrated optimal temperature at 37°C in constrast to PST_Mh_, which demonstrated lower activity at this higher temperature compared to other incubation temperatures. After 10 min incubation there is no longer an advantage for the activity at a higher temperature for these two enzymes. . Enzymes from three separate purification batches were tested in duplicate (*n* = 6).

We wanted to examine the performance of the three enzymes over a two hour time course at different incubation temperatures (25°C, 30°C, and 37°C) that may be required in downstream applications. We observed that all of the enzymes demonstrated a decrease in activity when measured throughout the time course as a result of the intrinsic labile nature of these enzymes, as well as the decrease in donor substrate and the changing nature of the acceptor molecule. Interestingly, higher incubation temperatures had different effects on the performance of the enzymes. At early time points (1–2 min) both PST_Nm_ and PST_Ec_ demonstrated optimal activity at 37°C, with decreased amounts of activity at lower temperatures (Figure 3). By 10 min (which was chosen because the majority of the drop in activity occurs by this time) the advantage imparted by the higher temperature was lost and the different temperatures gave similar activity values. In contrast, incubation of the reaction at 37°C was inhibitory to PST_Mh_, giving the lowest amount of activity compared to the other two temperatures. This trend continued throughout the course of the reaction. In addition to examining the activity at different temperatures we also looked at the percentage of activity remaining compared to the level at 1 min of incubation. In addition to having the highest activity of the three enzymes, PST_Nm_ had the highest percentage of activity remaining at all time points . For example at 10 min, the activity remaining for PST_Nm_ was 50.7% (25°C), 44.4% (30°C), and 34.7% (37°C). PST_Mh_ was intermediate in its stability with 33.8% (25°C), 30.3% (30°C), and 15.8% (37°C) of the activity was remaining at 10 min. PST_Ec_ was the most thermo-labile with only 26.9% (25°C), 20.4% (30°C), and 12.9% (37°C) of activity at 10 min.

### Donor Kinetics for CMP-Neu5Ac

In the development of glycosyltransfereases for synthetic purposes, one must consider the ability of an enzyme to use substrates efficiently. In the case of PSTs, this is a particularly relevant consideration since the donor molecular, CMP-Neu5Ac, is relatively expensive to produce and therefore could be a limiting reagent for synthesis.

Hence, we wanted to compare the kinetic parameters of the three bacterial PSTs for CMP-Neu5Ac rather than the acceptor molecule, since we did not anticipate the endogenous acceptor or our fluorescent synthetic acceptors to be used in downstream applications. PST_Mh_ was able to efficiently use CMP-Neu5Ac at lower concentrations than PST_Ec_ or PST_Nm_ when modifying the acceptor molecule GD3-FCHASE. Using concentrations of CMP-Neu5Ac ranging from 0.25 mM to 10–20 mM, PST_Mh_ was determined have a K_m_ of 1.10 mM compared to 5.67 mM and 4.93 mM for PST_Nm_ and PST_Ec_, respectively (Table 2). It is important to note that there is significant variation in the published K_m_ values for PST_Nm_ and PST_Ec_ depending on the assay, acceptor molecule, expression construct, and purification methods [21], [22], which is why testing the different enzymes in parallel is important for establishing their utility in downstream applications.

### PSA Chain Length on Synthetic Acceptors

**Figure 4:**
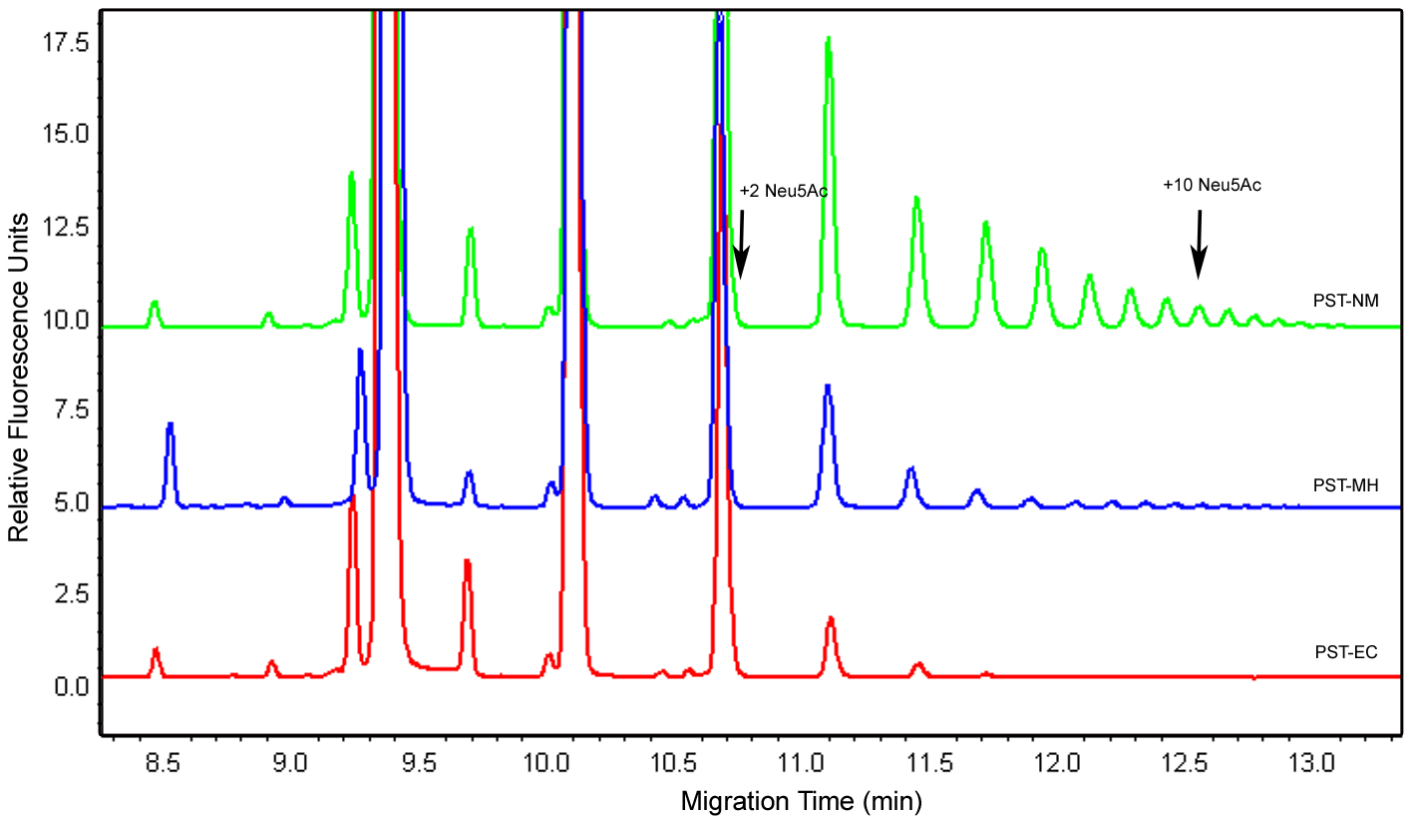
Analysis of PSA Chain Length by Capillary Electrophoresis. Capillary electrophoresis was used to analyze the reactions of GD3-FCHASE and CMP-Neu5Ac catalyzed by the three bacterial PSTs. Shown here are the reactions stopped at approximately 15% conversion. The large peak at ∼9.4 min represents the starting material and subsequent peaks are the products with additional Neu5Ac residues added. Acceptor plus 2 and 10 Neu5Ac products are indicated with arrows. The chain length of the products generated by PST_Nm_ (18 Neu5Ac) and PST_Mh_ (16 Neu5Ac) were significantly longer than that of PST_Ec_ (7 Neu5Ac). The electropherograms of the reactions have been overlaid on the same axis to aid in the comparison.

The ability to form different chain lengths may be needed for applications of these enzymes, such as therapeutic glycoprotein modification [26]. To determine product chain length heterogeneity we performed an analysis of the chain length distribution of the products generated from GD3-FCHASE at three different points during product formation: ∼15%, ∼30%, and ∼65% conversion. At all the stages of the reactions tested with the three enzymes, the predominant product is the acceptor plus one Neu5Ac residue. Each successive product is present in smaller amounts under these reaction conditions. Both PST_Mh_ and PST_Nm_ are capable of making PSA chains longer than twenty Neu5Ac residues in length, whereas PST_Ec_ makes short oligomers with a maximum length of ∼10 Neu5Ac. As can be seen in Figure 4, at 15% conversion PST_Nm_ and PST_Mh_ formed chains twice the length of that formed by PST_Ec_ (a total of 18, 16, and 7 Neu5Ac residues, respectively). This same trend was seen at higher percent conversions as well. The chain lengths of products formed by PST_Nm_ and PST_Mh_ increased to 22 and 18 at 30% conversion and then to chains longer than 20 Neu5Ac residues at 65% conversion. (It is difficult to determine the actual chain length of products longer than 20 Neu5Ac due to a limitation in the resolution of the products when analyzed by CE.) Unlike PST_Nm_ and PST_Mh_, the products formed by PST_Ec_ remained as short Neu5Ac oligomers at all percent conversions: 8 and 11 Neu5Ac, respectively, at 30% and 65% conversion. Interestingly at high product conversion (65%), the product with two Neu5Ac residues added represents a considerably higher proportion of the products formed by PST_Ec_ than the other two enzymes (∼30% of the total product compared to ∼15% for PST_Mh_ and less than 10% for PST_Nm_). In the case of PST_Mh_ and PST_Nm_ at the same stage of the reaction, the products longer than 6 Neu5Ac residues represent the majority of the products (∼40% for PST_Mh_ and ∼70% for PST_Nm_). This observation is interesting because it may be possible to use different enzymes in order to produce products with varying chain lengths and heterogeneity.

### Activity on Disialyated-Fetuin and PC12 Cells

**Figure 5:**
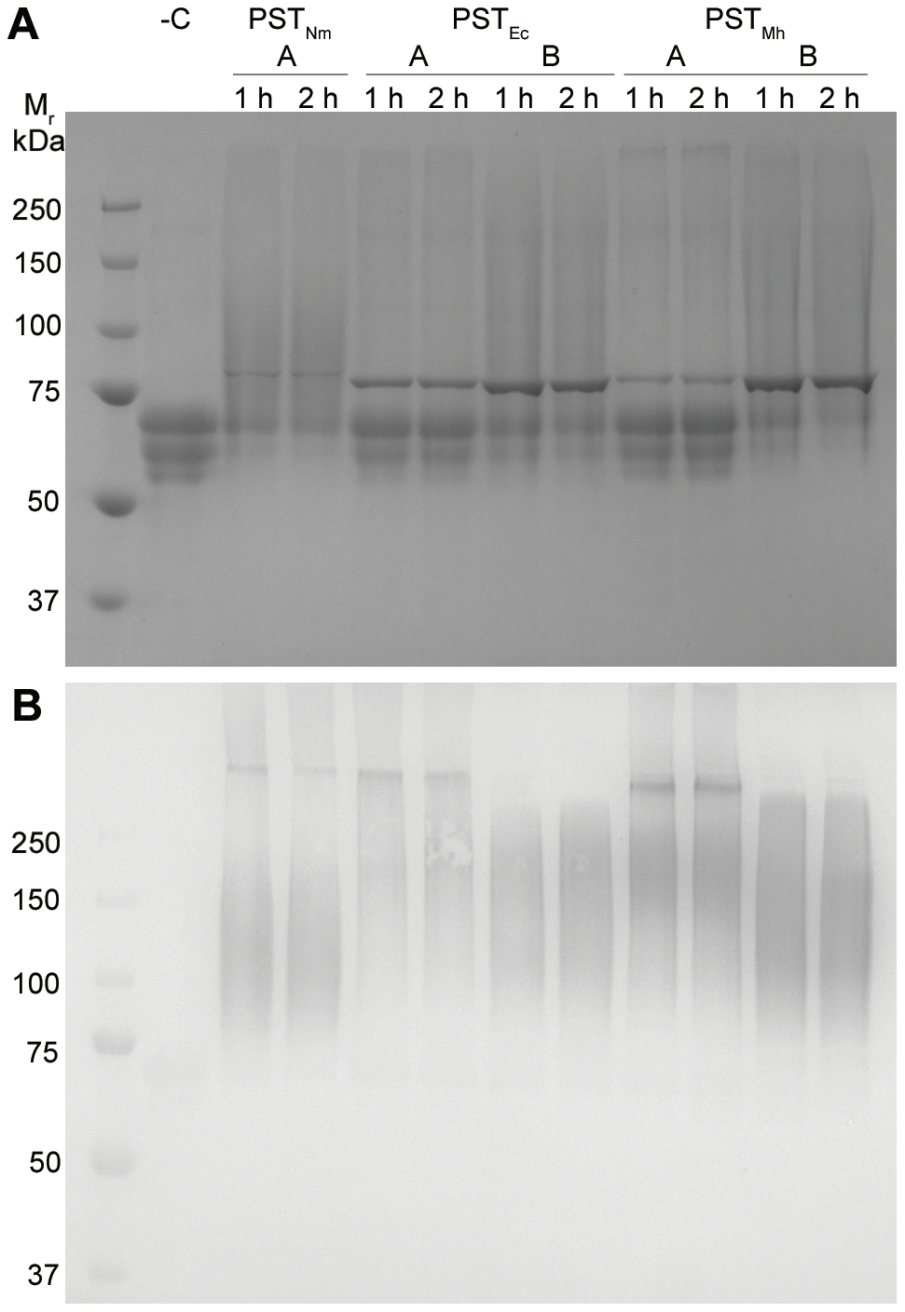
SDS-PAGE Analysis of PST Reactions on diSA-fetuin. A.The three PSTs were incubated with the acceptor molecule disialyl-fetuin using standard reaction conditions (lanes denoted “A”) as well as a reaction condition to improve the formation of the product (13 µg enzyme, lanes denoted “B”) for 1 or 2 h. A negative control reaction (containing no enzyme) was included (“-C”). The small band at ∼75 kDa indicates the PST added to the reaction. B. Following SDS-PAGE analysis, the proteins were transferred to a membrane and probed with α-PSA antibodies. All reactions were positive for PSA except the negative control lane containing unmodified disialyl-fetuin.

Recently we demonstrated the ability of PST_Nm_ to polysialylate therapeutic glycoproteins, which in turn resulted in an improved pharmacokinetic profile for the therapeutics [26]. Here we wanted to determine if PST_Mh_ is able to modify the model serum glycoprotein fetuin, which has been previously modified such that its glycans terminate in a disialyl moiety (diSA-Fet), in order to be a substrate for bacterial PSTs. When the three PSTs were incubated with diSA-Fet and CMP-Neu5Ac, all three enzymes were able to synthesize polysialylated material, as was evidenced by the change in migration of the product protein on an SDS-PAGE gel and was confirmed by a Western blot with anti-PSA antibodies (Figure 5). An examination of the intensity (based on gel densitometry) and molecular mass of the converted material on an SDS-PAGE gel showed that PST_Nm_ demonstrated the highest activity on this protein (69% conversion in 1 h). Under standard reaction conditions, both PST_Mh_ and PST_Ec_ were also able to modify diSA-Fet, however to a lesser degree (21% and 25.5% conversion, respectively). With all three enzymes, doubling the reaction time resulted in an increase of product formation. Next, the amount of PST_Ec_ and PST_Mh_ in the reaction was increased to ∼13 µg (chosen based on the maximal volume of enzyme that could be added to the reaction) in order to determine if it was possible to increase the amount of product formed. When additional enzyme was added to the reaction the amount of polysialylated product increased dramatically (39% conversion for PST_Ec_ and 63% for PST_Mh_).

**Figure 6:**
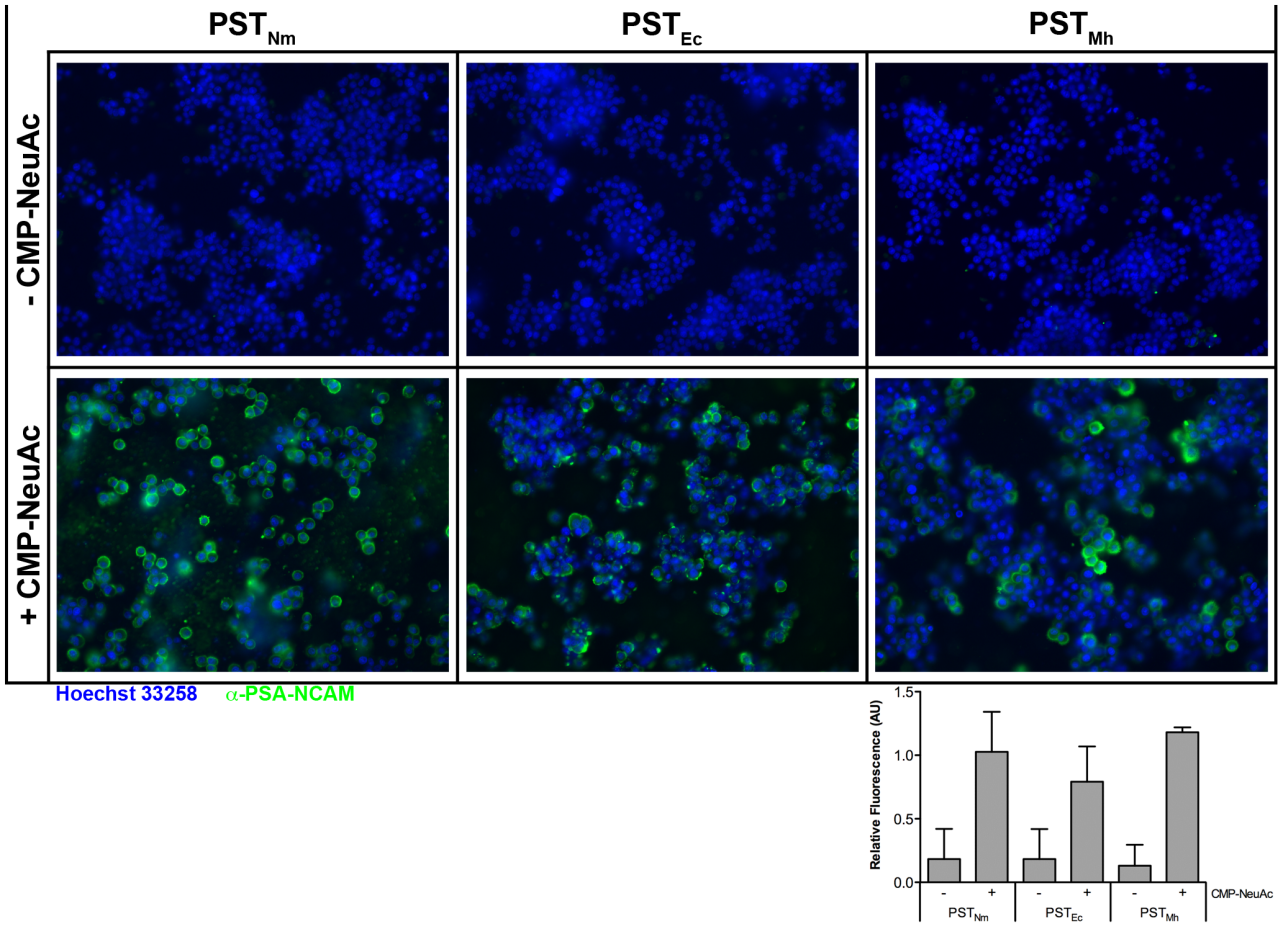
Polysialylation of cell-surface proteins on live PC12 cells. PC12 cells were incubated with the three PSTs in the absence and presence of 5 mM CMP-Neu5Ac for 1 hour at 37°C. Cell-surface PSA was detected using PSA-NCAM immunocytochemistry and nuclei were detected with Hoechst 33258 and confocal microscopy imaging. Following treatment of PC12 cells with all three PSTs in the presence of CMP-Neu5Ac specific cell-surface PSA labeling was detected (as indicated in the panels denoted “+CMP-Neu5Ac). Control reactions were also performed with the CMP-Neu5Ac absent; in these reactions PSA labeling was not detected. Quantitation of cellular polysialylation was performed using ImageJ (v 10.2) on the immunocytochemical images from two independent experiments by measuring the fluorescent intensity of 15 randomly selected cells per image. Images were pre-processed by performing background subtraction (50 pixel rolling ball radius) prior to measuring pixel intensity. Relative fluorescence values were calculated by dividing the PSA intensity (green fluorescence) by DAPI intensity (blue fluorescence). Data were normalized to average+CMP fluorescence for each experiment (n = 2).

In addition to the utility of bacterial PSTs in the modification of therapeutic proteins to improve pharmacokinetics, a recent application in tissue repair has been developed [31], whereby PST_Nm_ was used to synthesize PSA on the surface of cells in culture and in brain tissue. We therefore wanted to establish if PST_Mh_ was also capable of forming PSA on the surface of cells in culture. The three enzymes were incubated with CMP-Neu5Ac and the neuroprogenitor cell line PC12, which are known to over-express the native target of PST in mammalian systems, NCAM [32]. PSA production was detected by anti-PSA immunostaining and microscopy. In a live cell assay, all three enzymes were able to synthesize PSA on the surface of PC12 cells in the presence of CMP-Neu5Ac (Figure 6). Labeling in the absence of CMP-Neu5Ac was limited to levels comparable to non-specific binding of the secondary antibody (data not shown). Confocal microscopy confirmed that the PSA staining was restricted to the cell surface, consistent with the enzymes being excluded from entering live cells. Quantitation of PSA staining indicated that the three enzymes produced comparable levels of polysialylation under the experimental conditions tested (Figure 6 inset). As the bacterial PST enzymes require acceptor glycans terminating in a disialyl moiety, the present experiment indicated that PC12 cell surface proteins must contain this moiety. As was expected for a homogenous cell line culture, all cells were uniformly labelled by the PSTs. Interestingly, it was consistently observed that fewer cells were present on the coverslips treated with the PST enzymes, which is likely due to reduced adhesion of the PSA labelled cells. This observation is in line with the known effect of PSA on NCAM adhesion [1].

## Discussion

We have characterized the PST from the bacterium *M. haemolytica* A2 and compared several biochemical parameters to those of other glycosyltransferases of family GT-38. Based on the identification of this sequence and the following demonstration of PST activity, we propose that PST_Mh_ be classified as a member of family GT-38. This is the first GT-38 member that is not from either *E. coli* or *N. meningitidis* species.

The interest in using such PSTs for potential therapeutic applications requires that the enzyme of choice have qualities that are compatible with modification of high value therapeutic proteins, such as high purity and easy removal after modification. The production of high purity (recombinant) enzymes requires good over-expression, simple reproducible purification and good storage properties. The application of the enzyme requires that it be very efficient in conversion of the substrates that are used, so that the kinetic parameters should be such that large excesses of expensive substrates are not required for production. Because PSTs do not produce a single homogeneous product, it is also important to be able to consistently produce a product of desired length by changing the reaction conditions or by the generation of mutant enzymes with altered properties. With these parameters in mind as a starting point we sought to examine the performance of the three GT-38 polysialyltransferases.

We began the development of PST's for *in vitro* modification of glycoproteins and application to tissue engineering with the characterization of PST_Nm_ (expressed as the full-length version MalE-PST_Nm_) [22]. Our preliminary work with this enzyme showed that while the expression was adequate, the purification lead to material that was not as pure as needed for these applications, and that the enzyme had some stability issues that made it a good candidate for enzyme improvement. Next, we were able to show that truncation of the N-terminus of the protein greatly improved solubility and that an improved purification method could lead to higher purity, yields, and activity [26]. We also performed proof-of-concept experiments demonstrating that therapeutic proteins were modifiable by PST_Nm_ *in vitro*. The other well studied GT-38 enzyme from *E. coli* serotype K1 has also been applied to small scale glycolipid synthesis applications by others, but not as a pure protein or on protein or cell based acceptors [33]. While we are confident that glycolipids could be modified based on that work, we did not pursue gangliosides as acceptors as there is currently no demonstrated therapeutic need for these types of molecules. We have also shown that the application of PSTs to cell surface modification does not involve significant modification of glycolipids as the PSA formed on cell surfaces is completely removed by a trypsin treatment [31].

Having now made improvements to the issues of expression, solubility, purity, and utility of PST_Nm_, we wanted to address the same issues with PST_Ec_. While we were working on PST_Nm_ and PST_Ec_ we identified PST_Mh_ through genome mining, so that we were now able to test all three enzymes for the applications mentioned above, which is the basis for this study. It was readily apparent that all three enzymes could become more soluble when an N-terminal deletion was made between the enzyme and its fusion partner. The increase in expression was the greatest for the PST_Mh_ and in fact it yielded the highest level of active PST both in the soluble fraction and in terms of specific activity on the small synthetic acceptors. More specifically when PST_Mh_ was purified, over 21 mg of enzyme was recovered, in contrast to ∼4 mg of PST_Ec_ and ∼6 mg of PST_Nm_ from the same amount of cell extract (data not shown). Furthermore, the increase in yield came specifically from the protein of interest (and not any co-purified contaminant proteins) and therefore the degree of purity was also the highest for PST_Mh_. Despite the increased yield and purity, tests using diSA-Fet showed that it required larger amounts of PST_Mh_ to achieve the same level of protein modification with PST_Nm_, but unlike PST_Ec_, it could produce the same level of modification seen with PST_Nm_. Testing on the PC-12 neuroprogenitor cells showed that all three enzymes were capable of modifying cell surface proteins, with no overt differences in the ability to label proteins on the surface of live cells *in situ*, despite any differences in biochemical properties including (thermo)stability, product chain-length formation, and kinetics. Together the results of this study indicate that each of the three enzymes has specific qualities that may be useful in future therapeutic applications.

## Conclusions

As we have demonstrated that the GT-38 enzymes can be used to modify glycoproteins and mammalian cell surfaces, we are now tasked with improving them further for application in the production of therapeutics. We are planning to improve the specific activity, stability, and substrate range through directed evolution. It would be of interest to try to maintain the feature of the enzyme that enables it to form short chains of limited size distribution on glycoprotein acceptors, as that may be advantageous to applications requiring a more homogeneous polysialylation. The lower degree of stability at 37°C presents an ideal parameter to improve through directed evolution or site-directed mutagenesis, since any *in vivo* applications would require an enzyme optimally active at this temperature. In order to fully exploit these enzymes for *in vitro* synthesis we will improve the protein acceptor range of the enzyme as well so that more proteins/cells can be modified. In conclusion we have shown through a side-by-side comparison of three GT-38 polysialyltransferases that they have very different properties as recombinant proteins, and that the newest member from *M. haemolytica* A2 shows promise as a catalyst for the modification of therapeutic proteins and cell surfaces.
